# Surgery after treatment with imatinib and/or sunitinib in patients with metastasized gastrointestinal stromal tumors: is it worthwhile?

**DOI:** 10.1186/1477-7819-10-111

**Published:** 2012-06-15

**Authors:** Ronald Tielen, Cornelis Verhoef, Frits van Coevorden, Hans Gelderblom, Stefan Sleijfer, Henk H Hartgrink, Johannes J Bonenkamp, Winette T van der Graaf, Johannes H W de Wilt

**Affiliations:** 1 Department of Surgical Oncology, Radboud University Nijmegen Medical Center, Nijmegen, The Netherlands; 2 Department of Surgical Oncology, Erasmus Medical Center, Daniel Den Hoed Cancer Center, Rotterdam, The Netherlands; 3 Department of Surgical Oncology, The Netherlands Cancer Institute, Amsterdam, The Netherlands; 4 Department of Medical Oncology, Leiden University Medical Center, Leiden, The Netherlands; 5 Department of Medical Oncology, Erasmus Medical Center, Daniel Den Hoed Cancer Center, Rotterdam, The Netherlands; 6 Department of Surgical Oncology, Leiden University Medical Center, Leiden, The Netherlands; 7 Department of Medical Oncology, Radboud University Nijmegen Medical Center, Nijmegen, The Netherlands

**Keywords:** Gastrointestinal stromal tumors, imatinib, overall survival, progression-free survival, surgery, sunitinib

## Abstract

**Background:**

Standard treatment for metastatic gastrointestinal stromal tumors (GISTs) is systemic therapy with imatinib. Surgery is performed to remove metastatic lesions to induce long-term remission or even curation. In other patients, surgery is performed to remove (focal) progressive or symptomatic lesions. The impact and long-term results of surgery after systemic therapy have not been clearly defined.

**Methods:**

Between September 2001 and May 2010, all patients with metastatic GIST who underwent surgery for metastatic GIST after systemic therapy (that is, imatinib and sunitinib) at four Dutch specialized institutions were included. Primary end-points were progression-free survival (PFS) and overall survival (OS).

**Results:**

All 55 patients underwent surgery after treatment with systemic therapy. At the last follow-up, tumor recurrence or progression was noted after surgery in 48% of the patients who responded on systemic therapy, and in 85% of the patients who were treated while having progressive disease. Median PFS and OS were not reached in the group of responders. In the non-responders group PFS and OS were median 4 and 25 months, respectively. Response on systemic therapy and a surgical complete resection were significantly correlated to PFS and OS.

**Conclusions:**

Surgery may play a role in responding patients. In patients with progressive disease, the role of surgery is more difficult to distinguish in this retrospective analysis since PFS is short. Which patients benefit and whether this improves long-term outcome should be established in a multicentric randomized trial.

## Background

Gastrointestinal stromal tumors (GISTs) are the most common sarcoma of the gastrointestinal tract, accounting for <1% of all malignancies of the gastrointestinal tract [1,2]. Tumor size, perioperative tumor rupture and incomplete resection are factors known to influence development of metastases after surgery [3-5]. Metastases are mainly located in the liver and intraperitoneum [6]. Conventional chemotherapy has been ineffective in treating metastasized GIST with disappointing response rates below 10% [7].

Up to 85% of GISTs have activating mutations in tyrosine kinase receptor (KIT) and platelet-derived growth factor receptor alpha (PDFRA) genes, which are responsible for tumor development [8,9]. Imatinib, a selective inhibitor of several tyrosine kinases (for example, c-KIT and PDGFRA), has provided a generally safe and well-tolerated first-line therapy for patients with primary unresectable and metastatic GIST. Stable disease or tumor shrinkage is achieved in the majority of these patients [7,10,11]. Although most patients initially benefit from imatinib, its response is not maintained indefinitely as resistance commonly develops with a median time to progression of 18 to 24 months. After 5 years, most patients develop progressive disease which indicates that almost all patients will acquire resistance to imatinib [7,12,13]. Sunitinib, another oral multitarget tyrosine kinase inhibitor, has proven to be efficacious in patients intolerant or refractory to imatinib [14]. As a consequence of the introduction of effective systemic therapy (that is, imatinib and sunitinib), the median survival for metastatic GIST patients has substantially improved and is currently 5 years or more [15].

Prior to the introduction of systemic therapy, surgery was frequently applied. Outcomes were poor with a median survival of approximately 10 to 20 months and a 5-year survival of <10% for patients with metastasized GIST [3,16-18]. After the introduction of imatinib and sunitinib, surgery is now sometimes applied in patients with metastasized GIST. In patients who respond well to systemic therapy, the rationale for combining it with surgery is that by reducing tumor load, the risk of resistance to systemic therapy may be lowered. Observations from the phase III study comparing imatinib, 400 versus 800 mg daily, supports this approach to resect residual disease in an attempt to postpone secondary resistance [19]. Surgery is also applied in case of symptomatic or single progressive lesions. Others have already reported long-term progression-free survival (PFS) and overall survival (OS) after surgery in patients with metastatic GIST treated with imatinib [20-25]. This study aims to retrospectively describe the feasibility and outcome of surgery in a relatively large group of patients with metastatic GIST who underwent surgery after systemic therapy.

## Methods

### Patients and preoperative treatment

All patients in this study were referred to four Dutch institutions (The Netherlands Cancer Institute, Amsterdam; Leiden University Medical Center, Leiden; Radboud University Nijmegen Medical Center, Nijmegen; Daniel Den Hoed Cancer Center, Rotterdam) for treatment of metastatic GIST between September 2001 and August 2011. Each patient was evaluated in a multidisciplinary tumor board before the start of treatment with imatinib. All patients had a previous histopathologically confirmed diagnosis of GIST. Patient-, tumor- and treatment-specific data were extracted from sarcoma databases, medical records and patient charts at each institution. Recorded data included initial presentation, diagnosis, details of operations for primary and/or metastatic disease prior to systemic therapy, date of start of systemic therapy, duration and dose of systemic therapy, complications on systemic therapy, response to systemic therapy at time of surgery, date of surgery, type of surgery, completeness of resection, postoperative complications, postoperative systemic therapy, recurrence after surgery, last follow-up and disease status at last follow-up and, if applicable, date of death.

Before the start of imatinib, all patients had a baseline computerized tomography (CT) scan, and patients were clinically and radiographically re-evaluated every 1 to 6 months until surgery. Response to systemic therapy was classified as a complete response (CR), a partial response (PR), stable disease (SD) or progressive disease (PD) according to Response Evaluation Criteria in Solid Tumors (RECIST) [26]. For the purpose of analysis, patients were subdivided into two groups depending on their response on systemic therapy at the time of surgery. The responders group comprised patients with CR, PR and SD, and the non-responders group comprised patients with PD.

### Surgery and postoperative treatment

Surgical procedures were performed at all four institutions after a patient tailor-made decision was made in multidisciplinary tumor boards. The results of surgery were recorded as macroscopically complete (R0), macroscopically complete with positive microscopic margins (R1) or macroscopically incomplete (R2), based on surgical and pathological evaluation. Patients restarted systemic therapy depending on completeness of resection and preference of the treating physicians. Status of disease at last follow-up was determined using the most recent clinical evaluation. If a patient had deceased, date of death and disease status at death was recorded.

### Endpoints and statistics

PFS and OS were estimated using the Kaplan-Meier method. PFS was defined as the time from date of surgery after systemic therapy to date of first documented progression of residual disease, recurrent disease, metastatic disease or death from any cause. OS was defined as the time from date of surgery after systemic therapy to date of death from any cause. Statistical analysis was performed using SPSS statistical software, version 18.0 (IBM SPSS Software, New York, United States).

## Results

### Patients and preoperative treatment

Between September 2001 and May 2010, 55 patients with metastatic GIST underwent surgery after systemic therapy. Follow-up data was available until August 2011. Median age was 54 (range 18 to 77) years at diagnosis of metastatic GIST and 57 (range 20 to 80) years at time of surgery after systemic therapy. All patients had a GIST confirmed by experienced sarcoma pathologists at the four centers, and 52 patients (95%) were characterized by a positive c-KIT expression. Mutation status was available in 33 patients (60%); in 22 patients (40%) it was not routinely performed. Details on GIST manifestation are shown in Table 1. Thirty-one patients underwent one to three operations for primary or metastatic GIST before the start of systemic therapy for metastasized GIST.

**Table 1:** Gastrointestinal stromal tumor manifestation before start of systemic therapy

	**n = 55**
Synchronous disease	n = 21
Stomach + hepatic metastasis	6
Small intestine + hepatic metastasis	2
Colon + hepatic metastasis	1
Stomach + peritoneal metastasis	5
Duodenum + peritoneal metastasis	2
Small intestine + peritoneal metastasis	3
Rectum + peritoneal metastasis	1
Colon + hepatic and peritoneal metastasis	1
Metachronous disease	n = 34
Peritoneal metastasis	18
Hepatic metastasis	13*
Peritoneal and hepatic metastasis	3
Mutation analysis	n = 33
KIT exon 9 +/− exon 17	6
KIT exon 11 +/− exon 17	16
KIT exon 9 +/− exon 11	1
KIT exon 13	2
KIT exon 17	1
PDGFRA exon 12	1
PDGFRA exon 18	1
wildtype	5

*One patient had concurrent hepatic metastasis and metastasis in left vastus medialis muscle. KIT, tyrosine kinase receptor; PDGFRA, platelet-derived growth factor receptor alpha.

All patients initially received imatinib in a 400 mg daily dose. Twenty patients experienced complications on imatinib and one patient temporarily interrupted treatment because of gastrointestinal complications. Seventeen patients experienced PD from the start and imatinib was doubled to 800 mg daily. Eleven of these seventeen patients again experienced PD and switched to sunitinib 50 mg daily for 2 weeks followed by 1 week off-drug or a 37.5 mg daily continuous scheme. Five patients experienced complications on sunitinib: four patients experienced neurological and hematological complications, and sunitinib was lowered to a 37.5 mg daily continuous dose;one patient experienced complications and PD on sunitinib as well and switched back to imatinib 800 mg daily. All 55 patients underwent surgery after a median of 16 (range 3 to 72) months of systemic therapy. Systemic therapy was continued in all patients until surgery.

### Surgical outcomes and postoperative treatment

In the responders group (n = 35), 2 patients had a CR, 25 patients had a PR and eight patients had SD before surgery was performed after a median of 14 (range 3 to 72) months of systemic therapy. In the non-responders group (n = 20), 13 patients initially experienced PR or SD on systemic therapy before PD became apparent, and 7 patients experienced PD from the start. Surgery was performed after a median of 22 (range 5 to 72) months in this group.

Forty-five patients on imatinib underwent surgery and ten patients on sunitinib underwent surgery. Most surgical procedures were multivisceral resections (Table 2), and no tumor rupture occurred during surgery. A R0 resection was possible in 20 (57%) and 9 (45%) patients in the responders group and non-responders group, respectively. One patient in the responders group underwent a R0 resection of the left vastus medialis muscle because of an unusual metastasis at this location. Thirteen patients (37%) in the responders group and six patients (30%) in the non-responders group underwent a R1 resection. A R2 resection was performed in two patients in the responders group (6%), and in five patients (25%) in the non-responders group. In six of these patients, surgery could not be completed because of perioperative findings (that is, unresectable tumor). Of these six surgical interventions, one patient also had significant bleeding forcing the surgeon to terminate the surgical procedure. One patient underwent an emergency operation because of a bowel perforation and showed an unresectable tumor during surgery. The perforation was closed and a colostomy was performed. Surgical complications occurred in 11 patients. Four patients required a reoperation: for postoperative bleeding (n = 2), bile leakage (n = 1), and fascial dehiscence (n = 1). No patient died within 30 days of surgery. Systemic therapy was restarted after surgery in 27 patients (77%) in the responders group and in 19 patients (95%) in the non-responders group depending on expert opinion (that is, tumor board decision), remaining disease and resection type.

**Table 2:** Operative Procedures

Procedures*	**Number or%**
Gastrectomy + splenectomy + pancreatectomy ± omentectomy	6
Gastrectomy + splenectomy ± hepatectomy	3
Gastrectomy + splenectomy + pancreatectomy ± debulking†	2
Gastrectomy ± RFA	2
Small bowel resection + omentectomy ± sigmoid resection	9
Small bowel resection + colectomy + omentectomy ± RFA	3
Small bowel resection + duodenal resection	1
Omentectomy + abdominal wall	1
Peritonectomy stomach + debulking†	1
Hepatectomy ± RFA ± abdominal wall	10
Rectosigmoid resection	1
Debulking†	9
Resection left vastus medialis muscle	1
Exploratory laparotomy‡	6

*Twenty patients had a multivisceral (partial) resection during surgery; †Includes resection of multiple intra-abdominal laesions; ‡One patient underwent an emergency operation. RFA, radiofrequency ablation.

### Progression-free and overall survival

Complete follow-up data were available for 53 patients, with a median postoperative follow-up time of 41 (range 2 to 97) months. Two patients were lost to follow-up because they moved to another country. Tumor recurrence or progression after surgery was noted in 16 patients (48%) in the responders group and in 17 patients (85%) in the non-responders group. Disease-related death occurred in 9 patients (28%) in the responders group and in 14 patients (70%) in the non-responders group.

**Figure 1:**
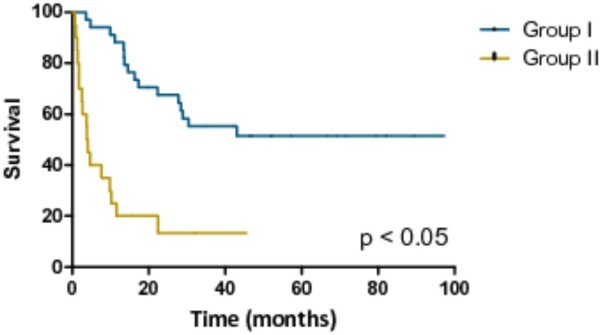
Progression-free survival (PFS) based on response to systemic therapy at the time of surgery, calculated from date of surgery.

**Figure 2:**
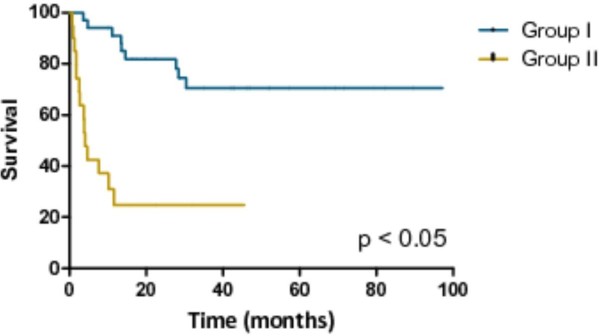
Overall survival (OS) based on response to systemic therapy at the time of surgery, calculated from date of surgery.

PFS and OS from time of surgery for both groups are shown in Figure 1 and Figure 2. Median PFS has not been reached in the responders group; in the non-responders group PFS was 4 (range 1to 27) months. One-, three- and five-year PFS was 85%, 55%, and 55%. For the non-responders group, the 1-, 3- and 5-year PFS was 22%, 13%, and 0%. Median OS has not been reached in the responders group; in the non-responders group OS was 25 (range 3 to 58) months. In the responders group, the 1-, 3- and 5-year OS was 100%, 78%, and 78%. In the non-responders group, the 1-, 3- and 5-year OS was 65%, 26% and 0%.

Univariate analysis (Table 3) of patient demographic, tumor, and treatment variables demonstrated response on systemic therapy and complete resection as prognostic factors correlating with PFS and OS. Patients responding to systemic therapy (that is, CR, PR or SD) at the time of surgery had a better outcome in terms of PFS and OS. The magnitude of this association is rather large with hazard ratios of 7.95 and 11.45. A complete resection was associated with a better PFS. Multivariate analysis yielded no significant outcomes due to relatively small numbers of patients in each cohort.

**Table 3:** Univariate analysis of tumor and treatment characteristics on progression-free and overall survival

	**PFS Hazard ratio (95% CI)**	**p-value**	**OS Hazard ratio (95% CI)**	**p-value**
Age at surgery		0.68		0.87
<60 years* (n = 31)	1		1	
>60 years (n = 24)	1.15 (0.58 - 2.29)		1.07 (0.47 - 2.43)	
Gender		0.19		0.68
Female* (n = 20)	1		1	
Male (n = 35)	0.63 (0.32 - 1.23)		0.68 (0.30 - 1.55)	
Response†		<0.05		<0.05
Yes* (n = 35)	1		1	
No (n = 20)	5.01 (2.46 - 10.22)		6.81 (2.83 - 16.38)	
Resection		<0.05		0.06
Complete* (n = 29)	1		1	
Incomplete (n = 26)	2.44 (1.20 - 4.96)		2.28 (0.98 - 5.28)	
Adjuvant therapy		0.81		0.69
Yes* (n = 46)	1		1	
No (n = 9)	0.89 (0.34 - 2.31)		1.28 (0.38 - 4.32)	
Location metastasis‡		0.52		0.57
Abdominal* (n = 33)	1		1	
Liver (n = 22)	0.79 (0.45 - 1.40)		1.21 (0.63 - 2.30)	

*Reference group; †response on systemic therapy; ‡patients with both liver and abdominal metastasis were grouped together in the abdominal group; ||surgery before start of systemic therapy. CI, confidence interval; OS, overall survival; PFS, progression-free survival.

## Discussion

In this study, we analyzed the feasibility and outcome of a large group of patients with metastatic GIST who underwent surgical resection after neoadjuvant treatment with imatinib and/or sunitinib. Surgery was performed to remove metastatic lesions to induce long-term remission or even curation in selected patients with an excellent response after systemic therapy, while in other patients surgery was performed to remove (focal) progressive symptomatic lesions.

Imatinib as the first-line systemic treatment in patients with metastatic GIST induces regression or stabilization in over 80% of patients, and sunitinib can achieve responses in patients refractory to imatinib [11,14]. A complete response or sustained ongoing response on systemic therapy is rarely seen, and discontinuation of systemic therapy usually leads to recurrence or rapid progression of disease [27]. This knowledge supports continuing systemic therapy in patients with responsive or stable tumor clones [13,28]. Nearly one-third of the patients in this study experienced PD while on systemic therapy. In at least some of these patients, it can be explained by the imatinib refractory mutation (that is, KIT exon 9 mutation) or the heterogeneous nature of GIST itself. Response measurement in GIST by using RECIST alone, however, might not be the best available tool. Assessment of the response using density and/or smaller changes in size are more precise and likely to yield information on the response more quickly [29,30] can be useful when CT findings are inconsistent with clinical findings [31-33]. However, a ^18^FDG-PET scan was not always available in the past in this study.

In the pre-imatinib era, median survival was 19 months for metastatic disease and 12 months for local recurrence [17]. Median survival for metastatic GIST patients treated with imatinib has improved to 5 years or more [12,15]. Prolonged PFS and OS rates observed after imatinib therapy in patients with metastatic GIST have been the topic of investigation on the effect of surgery following systemic therapy. Several retrospective studies have already reported a favorable outcome for patients responding to systemic therapy undergoing surgery following imatinib therapy (Table 4). In this series, it is not possible to dissect the specific contribution of surgery to the survival rates given the lack of an appropriate control group. A phase III trial was conducted to randomize patients with metastatic GIST responsive to imatinib to either continue imatinib alone or imatinib plus early surgery. Unfortunately, this trial was stopped due to lack of accrual. Evidence should therefore be collected using multicenter cohort analyzes such as the present study.

**Table 4:** Outcome of patients treated with imatinib followed by surgery for metastatic gastrointestinal stromal tumor

**Author**	**Number of patients**	**FU (median/months)**	**Survival**
Bonvalot *et al*., 2006 [23]	17	32*	62% 2-year OS*
Raut *et al*., 2006 [24]	60	14.6*	1-year OS was 95% for SD, 86% for LP, and 0% for GP†
Rutkowski *et al*., 2006 [25]	29	12	89.6% alive at last FU
Andtbacka *et al*., 2007 [20]	11	30.7‡	100% alive at last FU
	24	11.8||	79% alive at last FU
Gronchi *et al*., 2007 [21]	27	29	100% alive at 1-year (RD)
	8	12	60% alive at 1-year (PD)
DeMatteo *et al*., 2007 [22]	40	15**	100% alive at 2-year RD; 36% 2-year FR; 36% 1-year MR
Mearadji *et al*., 2008 [34]	6	46	33% alive at last FU
Mussi *et al*., 2010 [35]	49	31††	5-year DSS 82.9% in group A;
	31	13††	5-year DSS 67.6% in group B
Raut *et al*., 2010 [36]	50	16.4‡‡	Median OS not reached for RD, 18.5 months for LP, 8.9 months for GP
Yen *et al*., 2010 [37]	35	37	2-year OS 69.6% for PR + SD; 2-year OS 48.4% for LP
Present study	55	28||||	5-year OS 78% RD, and 3-year OS 26% PD

*Median for locally advanced and metastatic/recurrent disease. † Patients were divided into three categories: SD, LP, and GP. ‡ Complete resection. || Incomplete resection. ¶ Patients were divided into two categories: RD and PD. ** Patients were divided into three categories: RD, FR, and MR. †† Patients were divided into two categories: patient with best clinical response (group A), and patients with focal progression (group B). ‡‡ Patients were divided into three categories: RD, LP, and GP. ||||Patients were divided into two categories: RD and PD. SD, stable disease; LP, limited progression; GP, generalized progression; FU, follow-up; RD, responsive disease; PD, progressive disease; FR, focal resistance; MR, multifocal resistance; DSS, disease-specific survival; OS, overall survival; PFS, progression-free survival; PR, partial response.

The present study demonstrates a clear improvement in PFS and OS when surgery is performed in patients with responsive disease on systemic therapy and a complete resection is related to an improved PFS. This is in accordance with other reports (Table 4). The survival rates of patients in the non-responders group are comparable to historical data of surgically treated patients before the introduction of c-KIT targeting agents [3,17]. Evidence from randomized clinical trials is lacking, and it has therefore been difficult to determine the duration of systemic therapy before surgery. Some patients were thus operated on when progression of disease on systemic therapy became apparent. This is reflected in both the duration of treatment before surgery and the short PFS in the non-responders group. Given the relatively limited survival after surgery in patients with progressive disease at the time of surgery, we do not recommend surgery in these patients unless there is an urgent indication (for example, bleeding or obstruction).

In general, surgery after systemic therapy of advanced GIST appeared to be feasible and is not associated with enhanced morbidity compared to patients undergoing surgery alone for GIST. The reported number of incomplete (R1/R2) resections in this study is high, which has been observed by others. This reflects the extensiveness of the disease and the need to carefully choose between potential treatment options in patients with metastatic GIST.

## Conclusions

In our experience, patients with recurrent and/or metastatic GIST should be referred to centers with significant experience in surgery of these patients. Evaluation in a multidisciplinary sarcoma tumor board provides the optimal strategy for medical and/or surgical treatment in multicenter trials. The results of this study indicate that surgery may have an important role in responding patients. In patients with progressive disease, the role of surgery is more difficult to discern from this retrospective analysis since PFS is short and surgery is probably only beneficial in symptomatic patients in good clinical condition.

## Abbreviations

CR, complete response; CT, computerized tomography; GIST, gastrointestinal stromal tumors; KIT, tyrosine kinase receptor; OS, overall survival; PD, progressive disease; PDFRA, platelet-derived growth factor receptor alpha; PFS, progression-free survival; PR, partial response; RECIST, Response Evaluation Criteria in Solid Tumours; RFA, radiofrequency ablation; SD, stable disease.

## Competing interests

The authors declare that they have no competing interests.

## Authors’ contributions

Study concepts and design: RT, JW. Data acquisition: RT. Data analysis and interpration: All authors. Statistical analysis: RT, JW. Manuscript preparation: RT, CV, JW. Manuscript review: All authors. All authors read and approved the final manuscript.
